# Double-Hit Primary Plasma Cell Leukemia with *IGH/MYC* and *IGH/CCND1* Translocations

**DOI:** 10.1155/2020/8811114

**Published:** 2020-12-18

**Authors:** Masato Yasumi, Takaya Endo, Hiroshi Sata, Takahiro Karasuno

**Affiliations:** ^1^Department of Hematology, Rinku General Medical Center, Izumisano, Japan; ^2^Division of Hematology, Department of Internal Medicine, Daini Osaka Police Hospital, Osaka, Japan

## Abstract

Primary plasma cell leukemia (pPCL) is an aggressive variant of multiple myeloma (MM). Immunoglobulin heavy chain (*IgH*) translocations are found in a majority of pPCL cases, supporting a central relation to pathogenesis of pPCL. However, two independent *IgH* translocations are barely detected at the onset of pPCL, and their significance is yet to be elucidated. Here, we report a case of an aggressive pPCL with simultaneous *IGH/MYC* and *IGH/CCND1* translocations. A 73-year-old man was referred to our hospital with back pain and diagnosed as having pPCL with more than 50% circulating plasma cells. Cytogenetic analysis revealed 47, Y, t (X; 8;14) (q24; q24; q32), t (11; 14) (q13; q32), and +18. *IGH/MYC* and *IGH/CCND1* translocations were confirmed by fluorescence in situ hybridization analysis. Bortezomib and dexamethasone treatment achieved rapid elimination of peripheral malignant plasma cells, and the patient maintained a partial response for 18 months. After biological relapse, he received salvage therapy with ixazomib, lenalidomide, and dexamethasone, followed by pomalidomide and dexamethasone, and exhibited stable disease for an additional 14 months. Although *IGH/MYC* translocation in association with dysregulation of antiapoptotic pathway leads to worse prognosis in lymphomas, the novel agent-based regimen showed good efficacy, suggesting that *IGH/MYC* plays a different role in the pathogenesis of MM. *IGH/CCND1* and *IGH/MYC* translocations may have contributed to abrupt onset of pPCL in this case.

## 1. Introduction

Plasma cell leukemia (PCL) is a rare and aggressive form of multiple myeloma (MM), and the incidence of PCL was reported to be less than 1% of MM cases [1]. PCL is classified into two clinical types: primary PCL (pPCL) and secondary PCL [2]. In the former type, presentation is de novo and the prognosis is poor, with median overall survival (OS) of 4 months due to being refractory to various chemotherapies [1].

Recurrent immunoglobulin heavy chain (*IgH*) gene translocations involving *CCND1*, *FGFR3* and *MMEST*, *CCND3*, *MAF*, and *MAFB* are important oncogenic pathways observed in about 40% of MM and in 87% of pPCL cases [3]. Among them, *CCND1* is the most prevalent partner of *IgH* translocation, detected in 20–71% of pPCL cases [4]. Because most of these primary *IgH* translocations are speculated to be mediated by errors in *IgH* class switch recombination during B cell maturation, they are considered to be an early oncogenic event [3]. On the other hand, *IgH* translocations that target *MYC* are thought to be a very late and progressive event in the course of MM and rarely identified at the onset of MM, especially in pPCL [5].

Here, we report a case of an aggressive pPCL with simultaneous *IGH/MYC* and *IGH/CCND1* translocations.

## 2. Case Report

A 73-year-old man was referred to our hospital with a history of back pain for one month. Laboratory findings were as follows: a white blood cell count of 8.9 × 109/L with 55.0% of atypical plasma cells, hemoglobin level of 8.1 g/dL, platelet count of 55 × 109/L, lactate dehydrogenase level of 326 IU/L, creatinine level of 1.03 mg/dL, and blood urea nitrogen of 30.6 mg/dL. IgG was elevated at 4847 mg/dL with low levels of IgA and IgM (31 mg/dL and 9 mg/dL, respectively), and free light chain (FLC) analysis demonstrated a slight increase in free *λ* chain: *κ* FLC of 3.1 mg/dL, *λ* FLC of 50.8 mg/dL, and *κ*/*λ* ratio of 0.06.

**Figure 1:**
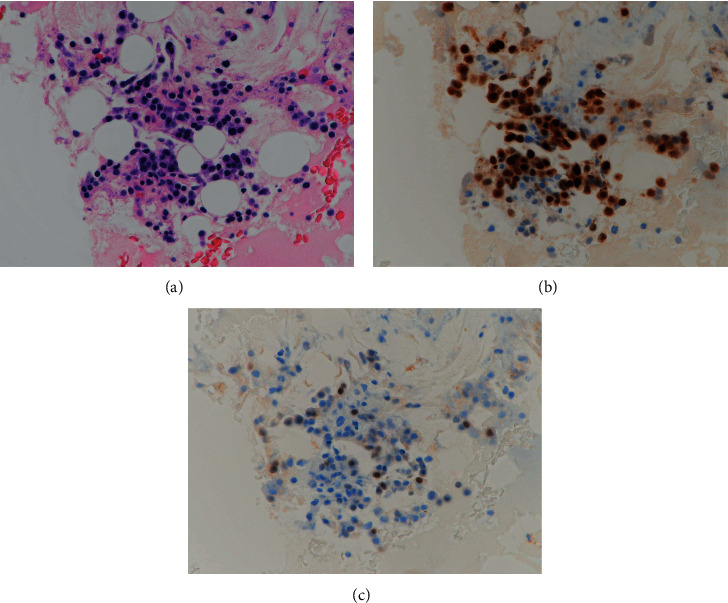
Pathological and immunohistochemical findings of bone marrow clot samples. (a) Hematoxylin and eosin staining showing infiltration of small-sized atypical plasma cells with eccentric nuclei. (b) Plasma cells were strongly positive for cyclin D1. (c) Plasma cells exhibited partial and weak MYC expression. Magnification: 400x.

**Figure 2:**
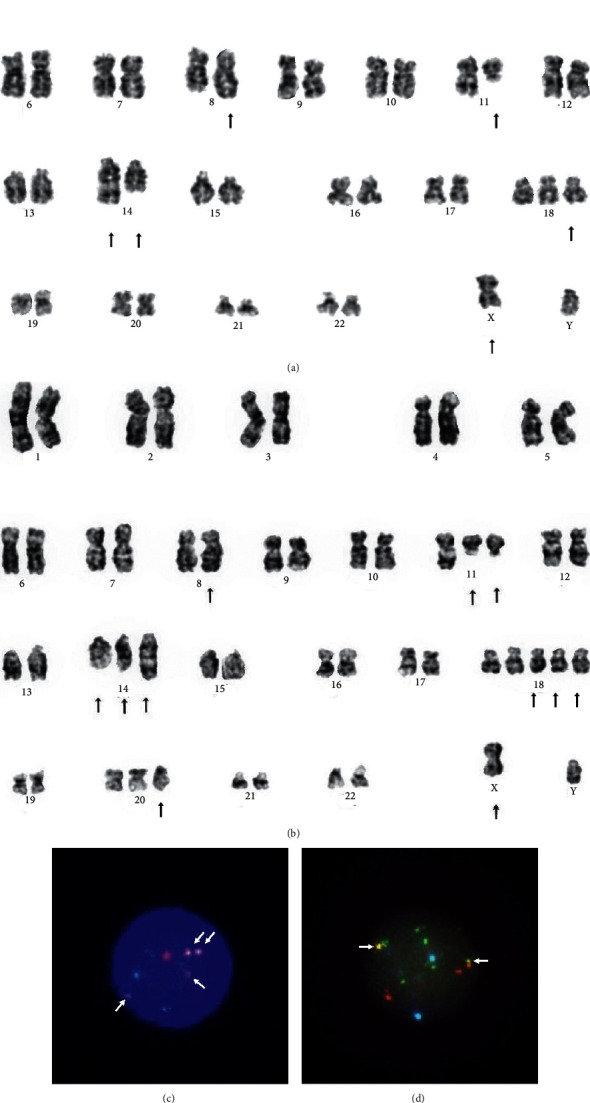
Karyotype of metaphase chromosomes and interphase fluorescence in situ hybridization (FISH) analysis obtained from the bone marrow. (a) The following complex karyotypes were observed at diagnosis: 47, Y, t (X; 8; 14) (q24; q24; q32), t (11; 14) (q13; q32), and +18. The arrows indicate the abnormal chromosomes. (b) Additional karyotype aberrations, including der (11)t (11; 14) and der (14)t (8; 14), were observed after IRd therapy. (c) FISH analysis was performed using a vysis LSI IGH/CCND1 dual color, dual fusion translocation probe. The orange signal represents CCND1, and the green signal represents IGH. Four fusion signals were observed. (d) FISH analysis was performed using a vysis LSI IGH/MYC/CEP 8 tri-color dual fusion probe. The orange signal represents MYC, the green signal represents IGH, and the aqua signal represents alpha satellite sequences on 8q11.1. Two fusion signals were visible.

Immunoelectrophoresis of serum identified IgG-*λ* monoclonal protein. Bone marrow examination revealed an infiltration of atypical plasma cells, comprising 55% of overall cellularity. Cytogenetic analysis revealed 47, Y, t (X; 8; 14) (q24; q24; q32), t (11; 14) (q13; q32), and +18 in 2 of the 20 cells studied. On immunohistochemistry, atypical plasma cells were strongly positive for cyclin D1 and partially weakly positive for MYC (Figure 1). We confirmed *IGH/MYC *and *IGH/CCND1* translocations by fluorescence in situ hybridization (FISH) analysis at the first relapse (Figure 2). Thus, he was diagnosed as having IgG-*λ* type primary plasma cell leukemia with *IGH/MYC *and *IGH/CCND1* translocations.

Initially, the patient was treated by Bd therapy (1.3 mg/m2 of bortezomib administered subcutaneously on days 1, 8, and 15 every 28 days, with 20 mg dexamethasone administered orally on days 1–2, 8–9, and 15–16). Malignant plasma cells rapidly disappeared from peripheral blood after one cycle of Bd therapy. He achieved a partial response after three cycles of Bd therapy. His IgG level was stable at around 1200 mg/dL for 18 months; however, it increased to 3083 mg/dL after 15 cycles of Bd therapy. As second-line therapy, IRd therapy (4 mg of ixazomib given orally on days 1, 8, and 15 every 28 days in addition to reduced doses of lenalidomide (10 mg daily on days 1–21) and 20 mg of dexamethasone weekly) was administered. IRd therapy was effective, and his IgG level decreased to 1380 mg/dL after five cycles. He achieved a partial response again. Unfortunately, he fractured the right femoral neck after 12 cycles of IRd therapy. He also developed severe pneumonia; therefore, we discontinued IRd therapy. Thereafter, bone marrow examination was performed, which revealed increased atypical plasma cells, and additional aberrations, including der (11)t (11; 14) and der (14)t (8; 14), were found on cytogenetic analysis (Figure 2(b) and Table 1). Due to his poor performance status and cytopenia, reduced-dose Pd therapy (1 mg of pomalidomide administered daily on days 1–14 every 28 days with 8 mg of dexamethasone weekly) was started. His IgG level had been stable at around 2000 mg/dL for 5 months. However, he developed recurrent infections and became bedridden, and then, he received palliative care and died 36 months after diagnosis.

## 3. Discussion

We report a case of pPCL harboring *IGH/MYC* and *IGH/CCND1* translocations at onset that showed a good response to novel agent-based treatment.

There are two main types of primary cytogenetic abnormalities in MM: hyperdiploidy and primary *IgH* translocations. In pPCL, *IgH* translocations were observed in 87%, and *CCND1* was the most common translocation partner observed in 20–71% [4], supporting a central role of *IGH/CCND1* translocation in the pathogenesis of pPCL. Recently, as high *BCL2*/*BCL-X*_*L*_ and *MCL-1* expression ratios were reported in t (11; 14) MM, *IGH/CCND1* translocation may contribute to plasma cell survival by altering the antiapoptotic pathway [7].

*MYC* is dysregulated or overexpressed in most human cancers and contributes to cell activation, cell proliferation, and apoptosis [8]. In MM, upregulation of *MYC* is involved in the progression from MGUS to MM [9] and also associated with leukemic and extramedullary presentation [10]. *MYC* is dysregulated by genetic rearrangements, such as translocations and amplifications, and is also modulated by deregulation of upstream pathways such as IRF4, DIS3/LIN28B/let-7, or MAPK [11].

Ig translocation with *MYC* is speculated to be a late and progressive event [12] and has been reported in 2.4% of newly diagnosed MM cases and rarely reported at the onset of pPCL [5]. To our knowledge, double translocations of *IgH* with *MYC *and *CCND1* were reported in only six cases of newly diagnosed MM, and this is the second case in pPCL [5, 13–16]. Nakayama et al. reported a 76-year-old patient with double-hit nonsecretory pPCL who had advanced disease [16], and Ji et al. described a 51-year-old patient with double-hit IgD myeloma who exhibited an aggressive clinical course and became refractory to conventional chemotherapy 18 months after the diagnosis [13]. However, there was no description of the detailed clinical course in other previously reported myeloma cases. Therefore, this is the first report of a clinical response to novel agent-based treatment.

Double-hit lymphoma with rearrangements of *MYC* and *BCL2* is recognized as a distinct entity under the WHO 2016 and known to have very poor prognosis [17]. Concomitant dysregulation of antiapoptotic molecule *BCL2* may counteract the proapoptotic function of *MYC*, resulting in rapid cell growth and resistance to therapy [18]. However, the prognosis of a double-hit case with rearrangements of *MYC* and *CCND1* in pPCL is not known, especially when treated with novel agent-based regimens. Despite leukemic presentation, frailty, as well as *MYC* and *CCND1* rearrangements, doublet and triplet regimens of novel agents had good efficacy in the patient and responded to treatment for more than 30 months after diagnosis. Recently, it was reported that *IGH/MYC* translocations in MM have extragenic *IGH* breakpoints, which are distinct from other B cell malignancies that usually have breakpoints in *IGH* switch regions [6]. These genetic alterations and cell differentiation states may contribute to affect the relatively good treatment response in *IGH/MYC-*possessing MM.

Interestingly, a previous study observed that cyclin *D* genes (cyclin D1, D2, and D3) were ectopically expressed in almost all MM cases from an early stage, implying a unifying and initial oncogenic event in MM [19]. In this case, juxtaposition of *CCND1* and *MYC* to the *IgH* gene enhancer may lead to simultaneous activation of initiating and progressive oncogenic pathways, resulting in abrupt onset of aggressive pPCL.

We also observed the accumulation of *IGH/MYC and IGH/CCND1* translocations over time during treatment, which suggested that *MYC* and *CCND1* signaling play a role in disease progression. There were discrepancies between cytogenetic analysis and FISH analysis in the time period and number of additional translocations. These may reflect the high sensitivity of FISH analysis in detecting chromosomal translocations [20].

Here, we described a case of double-hit primary plasma cell leukemia with *IGH/MYC* and *IGH/CCND1* successfully treated with novel agent-based regimens.

It is important to evaluate the *MYC* and *CCND1* signaling in the pathogenesis of pPCL for further understanding and identifying new therapeutic targets of pPCL.

## Figures and Tables

**Table 1:** Development of cytogenetic aberrations over time during treatment.

Period	Karyotype Result
At onset	47, Y, t (X; 8; 14) (q24; q24; q32), t (11; 14) (q13; q32), +18 [2]/46, XY [6]
At first relapse (after Bd therapy)	51, Y, t (X; 8; 14) (q24; q24; q32), −5, t (11; 14) (q13; q32), +12,−17, +18 + 20, +4mar [1]/46, XY[5]
After IRd therapy	52, Y, t (X; 8; 14) (q24; q24; q32), +der (11)t (11; 14) (q13; q32), t (11; 14), +der (14)t (8; 14) (q24; q32),+18, +18, +18, +20 [4]/46, XY [3]
